# A dataset representing the impact of aromatic nuclei on the carbon isotope of gases

**DOI:** 10.1016/j.dib.2019.105101

**Published:** 2020-01-03

**Authors:** Yanhong Liu, Yingqin Wu, Yanqing Xia, Lantian Xing, Chuntao Tian, Tianzhu Lei

**Affiliations:** a Gansu Provincial Key Laboratory of Petroleum Resources, Institute of Geology and Geophysics, Chinese Academy of Sciences, Lanzhou 730000, China; b University of Chinese Academy of Sciences, Beijing, 100049, China

**Keywords:** Aromatic nuclei, Carbon isotope, Natural gas, Pyrolysis experiment

## Abstract

This work presents a dataset which provides information on the influence of aromatic nuclei on the carbon isotope of gases. Gases analyzed herein were obtained by pyrolysis of model compounds and kerogens. The carbon isotope of gases from paraffin cracking with and without the addition of aromatic nuclei is summarized. We also obtained carbon isotope data for the gases from different type of kerogens, which indicate the role of aromatic nuclei in the formation of natural gases from kerogen cracking. Further interpretation and discussion of these data can be found in the related research article entitled “The methylation of aromatic nuclei — I: Implications for the geochemical evolution of gas” [1].

**Table:** Specifications Table

Subject	Geology
Specific subject area	Carbon isotope evolution of natural gases
Type of data	Table
How data were acquired	Carbon isotopic analyses were carried out on a Finnigan Mat Delta Plus isotope ratio mass spectrometer connected to an HP 5890II gas chromatograph.
Data format	Raw Analyzed
Parameters for data collection	The GC oven temperature was increased from 60 °C to 200 °C at 8 °C/min with initial and final hold times of 5 and 20 min, respectively. The conversion of individual compounds into CO_2_ was in a ceramic oxidation reactor at a temperature of 930 °C.
Description of data collection	The gas samples were prepared by pyrolysis of model compounds and kerogens in Pyrex glass tubes. They were subjected to GC-irMS to obtain their carbon isotopic compositions.
Data source location	The pyrolysis experiments and the GC-irMS analysis were performed at the Lanzhou Center for Oil and Gas Resources, Lanzhou, China.
Data accessibility	With the article
Related research article	Yanhong Liu, Yingqin Wu, Yanqing Xia, Lantian Xing, Chuntao Tian, Tianzhu Lei, The methylation of aromatic nuclei — I: Implications for the geochemical evolution of gas, International Journal of Coal Geology. https://doi.org/10.1016/j.coal.2019.103302 [1]

**Table:** 

**Value of the Data** • The carbon isotope analysis of several series gases provides foundation for detailed studies of the role of aromatic nuclei in the carbon isotope evolution of gases. • The information of the dataset could be used to study the mechanism of carbon isotopic fractionation of natural gases. • Another possible value of data could be to analyze the methylation processes of aromatic nuclei in the field of geochemical evolution of petroleum.

## 1 Data description

To understand the impact of aromatic nuclei and their methylation reactions on the carbon isotope of gases, several model compounds and kerogen samples were selected and subjected to pyrolysis experiments. The carbon isotope data of gases from four *n*-tricosane (*n*-C_23_) related systems are shown in Table 1, which indicate the influence of small aromatic nuclei with and without the catalysis of montmorillonite. Table 2 shows the isotopic compositions of gases from the pyrolysis of *n*-tetracosane (*n*-C_24_) in the presence of macromolecular aromatic nuclei and montmorillonite. The carbon isotope data of gases from different type of kerogens are summarized in Table 3, and they indicate the role of aromatic nuclei in the formation of natural gases from kerogen cracking. Additionally, the differences of isotopic fractionation between methane and ethane (δ^13^C_2_-δ^13^C_1_) are also shown in this paper.

**Table 1:** Carbon isotope data of the gases from the pyrolysis of the paraffin (*n*-C_23_) in the different reaction systems (*n*-C_23_, *n*-C_23_+N, *n*-C_23_+KSF, and *n*-C_23_+N + KSF).

	T (°C)	δ^13^C (‰)					δ^13^C_2_-δ^13^C_1_ (‰)
		CH_4_	C_2_H_4_	C_2_H_6_	C_3_H_6_	C_3_H_8_	
*n*-C_23_	370	−61.7	−29.9	−40.7	−25.5	−37.2	21.0
	390	−55.4	−25.3	−38.2	−17.1	−35.8	17.2
	410	−51.9	−24.9	−36.1	−15.1	−33.6	15.9
*n*-C_23_+N	370	−59.9	−28.4	−40.4	−31.0	−37.5	19.5
	390	−55.4	−25.4	−37.7	−21.1	−35.8	17.7
	410	−51.4	−26.0	−35.9	−17.7	−34.9	15.4
*n*-C_23_+KSF	350	−50.5	−30.4	−41.7	−22.0	−40.5	8.8
	360	−51.8	−32.9	−42.2	−18.8	−40.3	9.6
	370	−52.4	−30.5	−42.5	−20.8	−40.4	10.0
	380	−52.2	−29.5	−41.7	−18.1	−38.9	10.4
	390	−50.8	−32.1	−41.0	−9.9	−38.5	9.8
*n*-C_23_+N + KSF	350	−46.3	−29.1	−37.4	−19.3	−37.6	8.8
	360	−49.4	−34.0	−39.8	−21.7	−38.9	9.6
	370	−52.1	−31.7	−42.3	−19.2	−40.4	9.9
	380	−53.3	−29.9	−41.6	−24.0	−40.2	11.7
	390	−54.1	−27.1	−42.1	−17.2	−37.9	12.0

**Table 2:** Carbon isotope data of the gases from the pyrolysis of the paraffin (*n*-C_24_) in the presence of macromolecular aromatic nuclei and montmorillonite (*n*-C_24_+KSF + KEROGEN-500).

T (°C)	δ^13^C (‰)				δ^13^C_2_-δ^13^C_1_ (‰)
	CH_4_	C_2_H_6_	C_3_H_8_	*n*-C_4_H_10_	
340	−42.9	−36.7	−34.9	−33.6	6.1
360	−46.2	−38.0	−36.4	−34.6	8.2
380	−47.3	−40.1	−37.2	−34.3	7.2
400	−47.9	−39.4	−35.5	−33.0	8.5
420	−47.1	−37.0	−30.2	−17.0	10.1
440	−46.2	−33.0	−24.7	–a	13.3
460	−44.4	−30.6	–a	–a	13.9
480	−42.3	−26.5	–a	–a	15.8
500	−40.9	−21.6	–a	–a	19.3

a Not determined.

**Table 3:** Carbon isotope data of the gases from the pyrolysis of kerogen with and without montmorillonite.

	T(°C)	δ^13^C(‰)				δ^13^C_2_-δ^13^C_1_(‰)
		CH_4_	C_2_H_6_	C_3_H_8_	CO_2_	
TYPE-I	300	–48.6	–38.5	–35.9	–13.6	10.2
	350	–47.7	–37.6	–33.6	–11.5	10.1
	400	–47.1	–39.0	–36.5	–12.1	8.1
	450	–43.4	–35.3	–27.5	–14.0	8.1
TYPE-I+KSF	300	–48.5	–38.3	–36.0	–13.4	10.3
	350	–47.8	–37.3	–33.6	–14.2	10.6
	400	–44.8	–37.3	–34.9	–14.7	7.5
	450	–42.2	–35.0	–26.6	–15.0	7.2
TYPE-II/a	300	–33.4	–31.7	–31.4	–12.6	1.7
	350	–36.2	–32.2	–26.6	–14.0	4.0
	400	–34.2	–28.3	–25.8	–14.3	5.9
	450	–31.0	–26.3	–14.6	–14.1	4.7
TYPE-II/a+KSF	300	–32.9	–29.2	–28.7	–13.6	3.7
	350	–36.5	–29.3	–23.6	–15.2	7.2
	400	–34.0	–27.7	–23.8	–15.6	6.3
	450	–31.4	–20.5	–19.2	–16.8	10.9
TYPE-II/b	300	–39.7	–33.4	–31.0	–12.7	6.3
	350	–42.3	–33.2	–27.8	–14.0	9.1
	400	–38.2	–30.7	–27.6	–14.0	7.5
	450	–33.8	–27.7	–16.0	–15.1	6.1
TYPE-II/b+KSF	300	–38.6	–32.5	–29.7	–14.6	6.1
	350	–40.0	–31.5	–26.8	–16.6	8.4
	400	–36.6	–29.1	–24.1	–15.3	7.5
	450	–32.8	–20.1	–23.5	–17.1	12.7
TYPE-III	300	–34.2	–28.4	–26.5	–13.2	5.7
	350	–35.6	–27.2	–25.1	–12.5	8.4
	400	–33.4	–27.3	–24.8	–14.0	6.1
	450	–28.4	–22.5	–a	–14.5	5.9
TYPE-III+KSF	300	–33.4	–27.7	–25.1	–15.6	5.7
	350	–34.2	–26.4	–23.5	–16.3	7.8
	400	–34.1	–26.0	–22.4	–16.0	8.0
	450	–29.7	–18.7	–a	–16.8	11.0

a Not determined.

## 2 Experimental design, materials, and methods

### 2.1 Chemical and materials

Model compounds were selected to investigate the mechanism of effect of aromatic nuclei, while kerogens were selected to understand the action of aromatic nuclei in complex geological systems. Detailed information for model compounds and kerogens were described in the related research article [1]. Briefly, *n*-tricosane (*n*-C_23_), *n*-tetracosane (*n*-C_24_), naphthalene (N), and montmorillonite KSF were obtained from commercial suppliers and used as received. Four kerogen samples selected for pyrolysis experiments were prepared from the following rocks using the traditional HF–HCl approach [2]. The samples comprised the Green River formation shale from the Green River Basin (Type-I), the oil shale from the Minhe Basin (Type-II/a), the carbonaceous shale from the Yanqi Basin (Type-II/b), and the brown coal from the Huang County Basin (Type-III). Macromolecular aromatic nuclei (KEROGEN-500) were obtained by artificial maturation of the Type-II/b kerogen, which was performed in a closed system at 500 °C for 72 h to reduce its gas formation ability.

### 2.2 Pyrolysis experiments

1) The systems of *n-*C_23_/*n*-C_23_+N/*n-*C_23_+KSF/*n*-C_23_+N + KSF

The *n-*C_23_ and *n*-C_23_+N systems were design to investigate the influence of aromatic nuclei without a catalyst. They were pyrolyzed at 370, 390, and 410 °C. The *n*-C_23_+KSF and *n*-C_23_+N + KSF systems were used to understand the effect of aromatic nuclei under the catalysis of clay. The pyrolysis temperatures were 350, 360, 370, 380, and 390 °C. The *n*-C_23_+N + KSF system was taken as an example to illustrate the experimental procedures. A mixture of *n*-C_23_+N + KSF (300 mg:150 mg:1500 mg) (the dosage for other three systems was the same as the *n*-C_23_+N + KSF system) was added to the Pyrex glass tube (22-cm length, 1.3-cm internal diameter, and 0.2-cm thick). The tube was flushed with dry nitrogen five times to ensure the complete removal of air. It was then evacuated, and sealed. The tube was placed in Muffle furnace and heated at the desired temperature for a time period of 72 h at a heating rate of 2 °C/min. After cooling the tube to room temperature, it was placed in a vacuum line (pressure equal to 10^−5^ MPa). After isolating the extraction line from the vacuum pump, each tube was carefully broken into pieces to liberate the generated gases, which was pumped into a calibrated volume to quantify their total yield and recover them for molecular and isotopic analysis.

2) The system of *n*-C_24_+KEROGEN-500 + KSF

This system was design to investigate the role of macromolecular aromatic nuclei with montmorillonite. A mixture of *n*-C_24_/KEROGEN -500/KSF (300 mg:150 mg:1500 mg) was added to the Pyrex glass tube. They were pyrolyzed at 340, 360, 380, 400, 420, 440, 460, 480, and 500 °C for a time period of 72 h. The experimental process was the same as described above.

3) Kerogens and the mixtures of the kerogen and montmorillonite KSF

The kerogens as well as the mixtures of the kerogen and montmorillonite KSF (ratio of 1:2, usually 500 mg of kerogen with 1000 mg of mineral) were also sealed in the Pyrex tubes under vacuum. They were pyrolyzed at a temperature of 300, 350, 400, and 450 °C. The pyrolysis experiments were performed using the same procedures described above.

### 2.3 δ^13^C measurements of gaseous products

Compound-specific isotopic analysis of gaseous products was performed on a Finnigan Mat DELTA plus isotope ratio mass spectrometer (irMS) connected to an HP 5890II gas chromatograph (GC). The GC was equipped with a split/splitless inlet system and a 30 m × 0.32 mm i.d. HP-PLOT Q column (film thickness 0.25 μm; Agilent, USA). The GC oven temperature was increased from 60 °C to 200 °C at 8 °C/min with initial and final hold times of 5 and 20 min, respectively. The separated compounds were converted to CO_2_ by passing the eluting analyte stream through a ceramic oxidation reactor (with three braided NiO/CuO/Pt wires) at a temperature of 930 °C. The ^13^C of CH_4_ reference gas was monitored for every three samples to ensure that the precision and accuracy of the determination were maintained. The measurement precision was ±0.5‰ for δ^13^C.
